# Data for positive selection test and co-evolutionary analysis on mammalian cereblon

**DOI:** 10.1016/j.dib.2019.104499

**Published:** 2019-09-12

**Authors:** Wataru Onodera, Toru Asahi, Naoya Sawamura

**Affiliations:** a Faculty of Science and Engineering, Waseda University, TWIns, 2-2 Wakamatsu, Shinjuku, Tokyo, 162-8480, Japan; b Research Organization for Nano & Life Innovation, Waseda University, Japan

**Keywords:** Cereblon, E3 ubiquitin ligase, AMPK, Positive selection, dN/dS

## Abstract

Cereblon (CRBN) is a substrate recognition subunit of the CRL4 E3 ubiquitin ligase complex, directly binding to specific substrates for poly-ubiquitination followed by proteasome-dependent degradation of proteins. Cellular CRBN is responsible for energy metabolism, ion-channel activation, and cellular stress response through binding to proteins related to the respective pathways. As CRBN binds to various proteins, the selective pressure at the interacting surface is expected to result in functional divergence. Here, we present two mammalian CRBN datasets of molecular evolutionary analyses. (1) The multiple sequence alignment data shows that positive selection occurred, determined with a dN/dS calculation. (2) Data on co-evolutionary analysis between vertebrate CRBN and related proteins are represented by calculating the correlation coefficient based on the comparison of phylogenetic trees. Co-evolutionary analysis shows the similarity of evolutionary traits of two proteins. Further molecular, functional interpretation of these analyses is explained in ‘Positive selection of Cereblon modified function including its E3 Ubiquitin Ligase activity and binding efficiency with AMPK’ (W. Onodera, T. Asahi, N. Sawamura, Positive selection of cereblon modified function including its E3 ubiquitin ligase activity and binding efficiency with AMPK. Mol Phylogenet Evol. (2019) 135:78-85. [1]).

**Table:** Specifications Table

Subject area	Biology
More specific subject area	Molecular evolution
Type of data	Table, Figure
How data was acquired	Phylogenetic tree acquired using maximum likelihood & neighbor-joining method at MEGA7 software. dN/dS acquired using maximum likelihood & counting method at Selecton server and Datamonkey server. Degree of coevolution between 2 proteins acquired by mirror tree method at MirrorTree server.
Data format	Analyzed
Experimental factors	Nucleotide coding sequences were downloaded from NCBI GenBank.
Experimental features	Sequences were aligned using ClustalW at MEGA7. 1-to-1 orthologous relationship of sequences was checked using OMA database and Ensembl.
Data source location	Institution: NCBI GenBank (Data download source) City: Rockville Pike, Bethesda Country: USA
Data accessibility	Analyzed data only available with this article. Sequences used in this article available at NCBI GenBank (https://www.ncbi.nlm.nih.gov/) via accession number (see Supplementary Table 1).
Related research article	W. Onodera, T. Asahi, N. Sawamura, Positive selection of cereblon modified function including its E3 ubiquitin ligase activity and binding efficiency with AMPK. Mol Phylogenet Evol. (2019) 135:78-85 [1].

**Table:** 

**Value of the Data** • The positively selected (C366) of CRBN was detected as novel functional, experimentally confirmed site, which may be targeted as potential chemotherapeutic site as CRBN has potential to be the target molecule for therapy including multiple myeloma. • The selective pressure on mammalian CRBN was quantified by dN/dS; this provides evolutionary insights when a further residue-level study is conducted. • The co-evolutionary analysis of CRBN demonstrated the usefulness of the analysis of other CRBN-binding proteins of interest to understand the evolutionary relationships.

## 1 Data

**Fig. 1:**
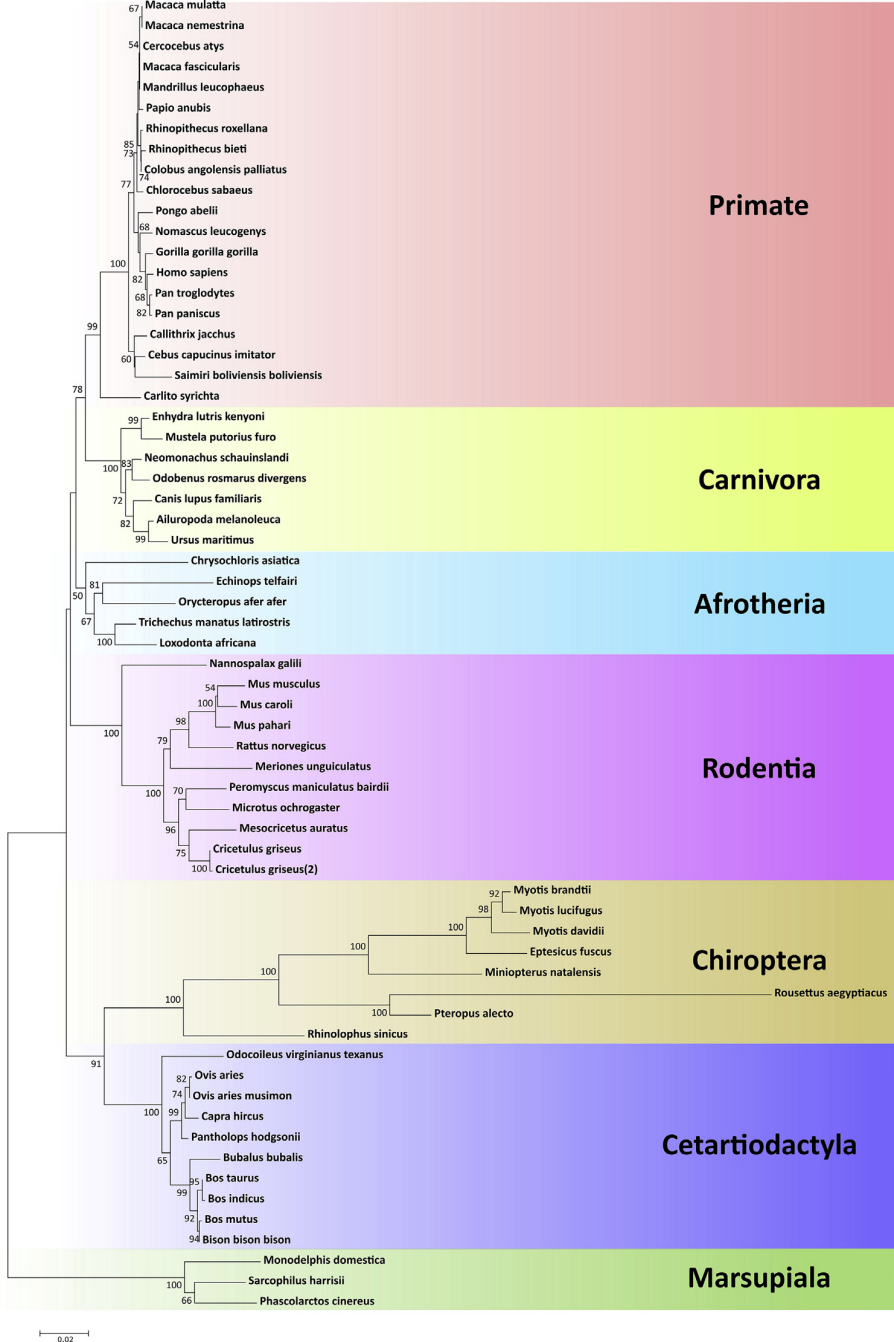
**Phylogenetic tree of full length CRBN tree**. The tree was constructed using the Neighbor-Joining method with bootstrapping (1000 replicates). The scale bar indicates the branch length scaled by substitution per site.

**Fig. 2:**
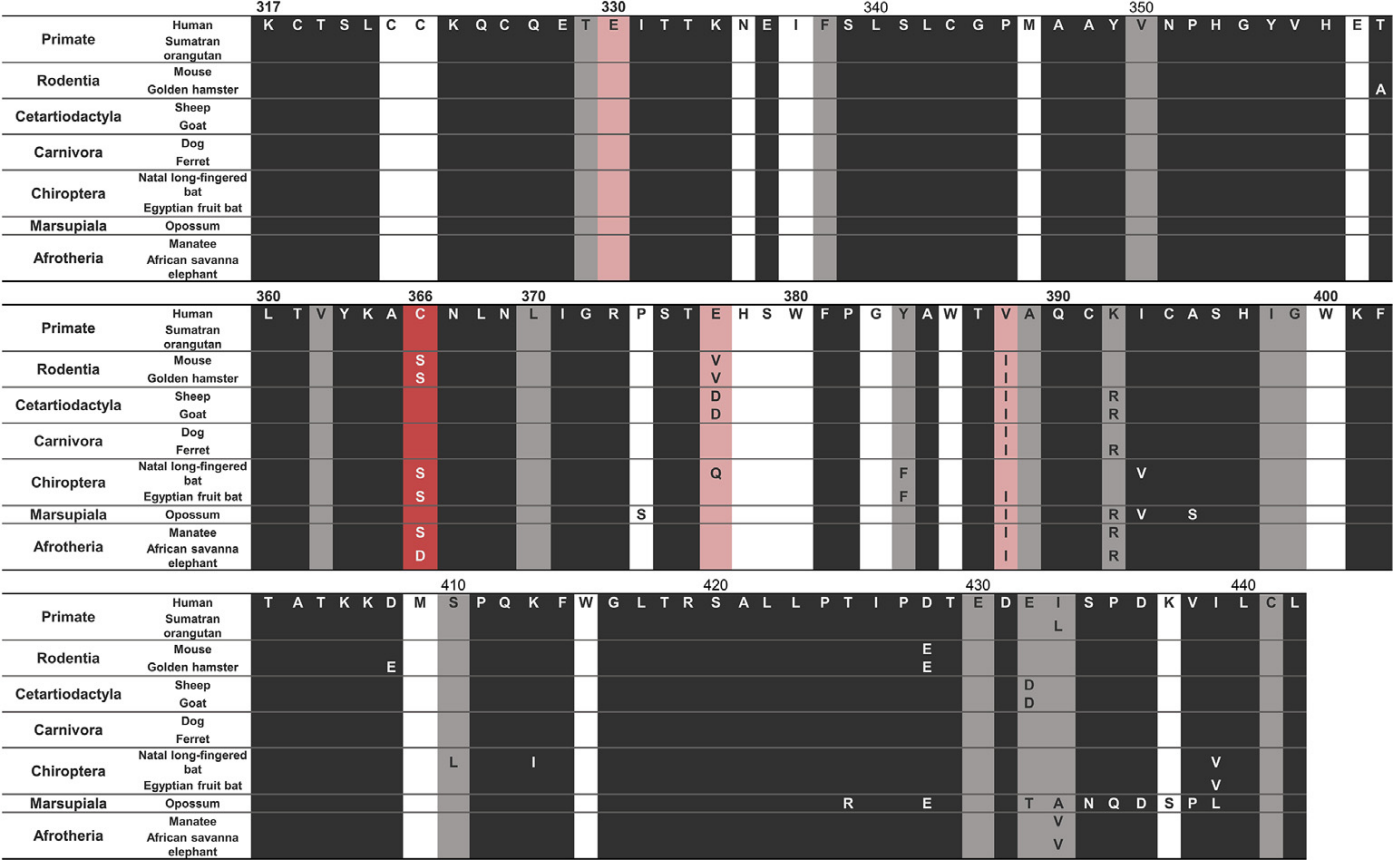
**Variation of amino acids and codon specific selection among mammalian CULT domain**. Sequences are based on Human CRBN and representative species are displayed from each clade. Columns with human only sequence letters indicate conserved sites, whereas the others show variations. Dark grey columns are inferred as negatively selected codons with a p-value<0.10 calculated by FEL. The dark red column shows a sign of positive selection with a p-value<0.10, also indicated in table 1bof [1]. Lighter grey and red columns indicate negative and positive selection with no statistical significance.

**Fig. 3:**
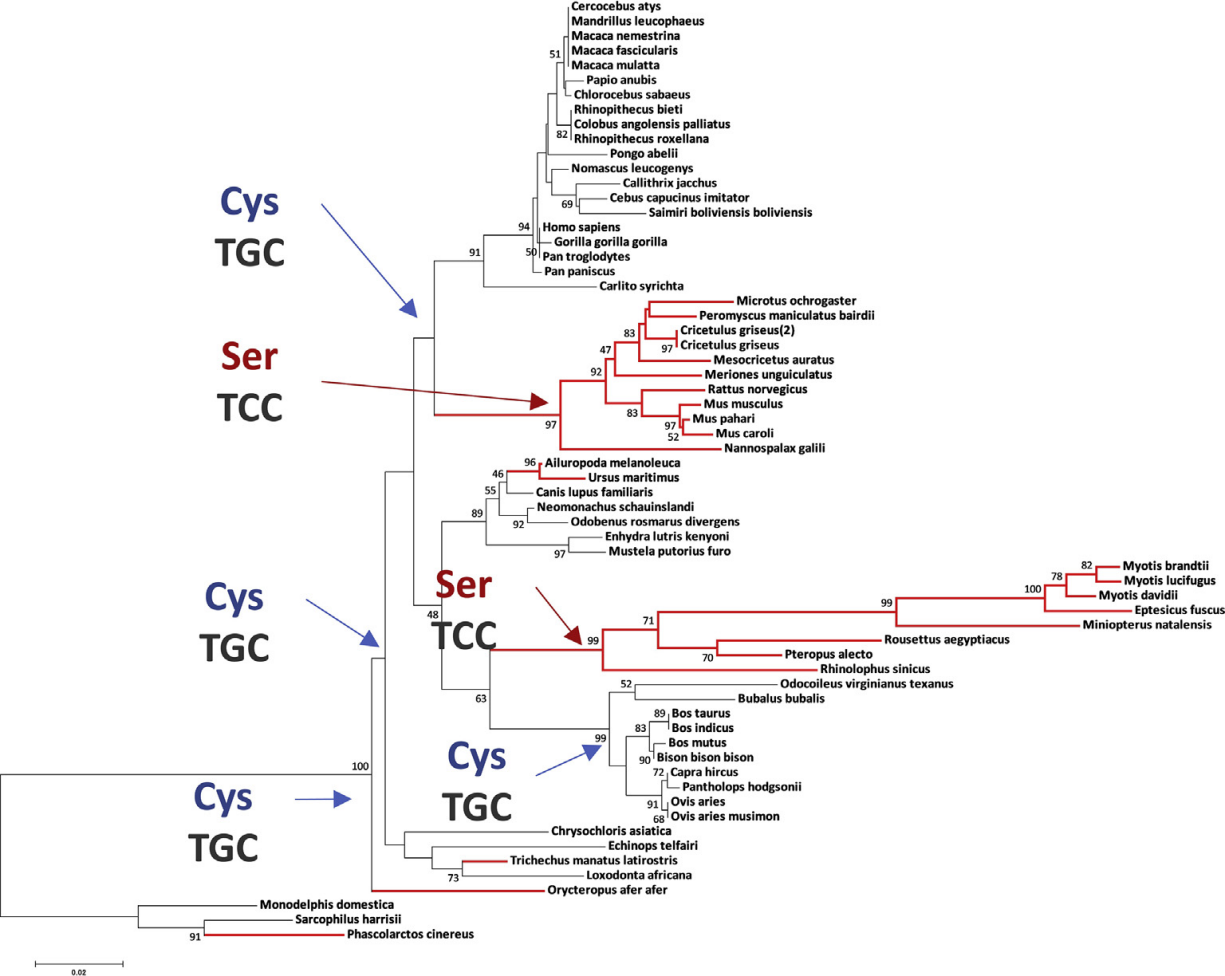
**Inferring ancestral sequence of codon 366 using CULT domain CRBN tree**. Ancestral sequence of mammalian CRBN was inferred using MEGA7. Codon shown in the tree indicates the ancestral codon of 366.

The data contains phylogenetically analyzed CRBN sequences. The sequences were collected from NCBI GenBank (sequence accession numbers available in Supplementary Table 1). Fig. 1 shows phylogenetic tree of the mammalian CRBN sequence reconstructed using maximum likelihood and neighbor-joining method. On the same dataset, site-model test for detection of positively selected site (position 366) was applied, represented in Fig. 2. Ancestral state of position 366 are illustrated in Fig. 3 estimated using maximum likelihood method. The result for coevolutionary analysis of vertebrate CRBN based on mirror tree method are listed in Table 1 and Fig. 3.

**Table 1:** Co-evolution analysis between domains of CRBN. Co-evolutionary signals between CRBN and its related proteins were calculated for Lon and CULT domain. As trend, LON domain exhibited larger co-evolutionary signals compared to CULT domain. AMPKɑ and Meis2 had statistically significant increase for Lon domain. (p-value with * < 0.10, ** < 0.05, *** < 0.01).

Protein	Symbol	Correlation coefficient			P-value (Lon vs CULT)
		Full length	Lon domain	CULT domain	
Complex factor	DDB1	0.892	0.856	0.792	0.363
	RBX1	0.847	0.773	0.748	0.734
Binding protein	AMPKα	0.935	0.935***	0.718	0.0001
	IKZF1	0.923	0.852	0.756	0.1442
	Meis2	0.922	0.911***	0.765	0.008
	SQSTM1	0.902	0.87*	0.757	0.075
	BK channel	0.841	0.816	0.669	0.101
Conserved protein	GAPDH	0.836	0.803	0.755	0.515
	GPI	0.857	0.798	0.833	0.555
	EF-1α	0.641	0.636	0.446	0.171
	β-Actin	0.673	0.606	0.681	0.516

## 2 Experimental design, materials, and methods

### 2.1 Data collection of sequences

Protein coding sequences of mammalian *crbn* genes were obtained from GenBank [2] in September 2017 (Supplementary Table 1). Partial sequences were excluded from the dataset. The sequences were aligned with ClustalW implemented in MEGA7 [3]. The default parameters were used for ClustalW. Redundant sequences were removed manually after multiple sequence alignment, 64 sequences were further analyzed (Supplementary Table 1).

Gene copy numbers were determined to validate the orthologous relationships of *crbn* genes. They were confirmed with the orthologous matrix (OMA) database and the orthologues view of Ensembl [4], [5]. A total of 42 sequences out of 64 were registered in those databases. Among the registered sequences, 41 species had a single copy of *crbn* (Supplementary Table 1).

### 2.2 Phylogenetic tree reconstruction

Phylogenetic trees of the CULT (cereblon domain of unknown activity, binding cellular ligands and thalidomide) domain (position of protein: 317-442), Lon domain (position of protein: 80-316) and full length *crbn* were constructed. Trees were built using maximum likelihood (ML) estimation implemented with MEGA7. The Kimura two parameter substitution model with discrete Gamma distribution of five categories were selected based on Akaike information criterion (AIC) scores [6]. The dataset was also analyzed using the neighbor-joining method in the Tamura three parameter substitution model [7], [8]. Bootstrap resampling was conducted 1000 times for each method (Fig. 1, fig1 in Ref. [1] for the CULT domain).

### 2.3 Positive selection test and ancestral sequence reconstruction

The Selecton server was used to identify positive selection using the site-model [9], [10]. Briefly, the server conducts likelihood ratio test (LRT) between the null hypothesis (M7 or M8a) that does not allow positive selection and the alternative hypothesis (M8) that allows positive selection (dN/dS > 1) to determine if there is positive selection in the dataset. The MEC (Mechanistic codon model), which assumes positive selection, uses AICc (AIC corrected) to compare the fitness in the dataset as it is not a nested model. If there is positive selection in the dataset, the Selecton server calculates dN/dS for each site and presents sites with a dN/dS statistically significant above one as positively selected site. A Bayesian approach was used for the dN/dS calculation. To assess the reliability of dN/dS values, a confidence interval defined by the 5th and 95th percentile of the posterior distribution is used. When the lower bound of the confidence interval is larger than one, the site is defined as positively selected site [10]. The dataset did not show statistical significance between M8 and M8a but showed statistical significance between M8 and M7. MEC fitted the dataset best as it had the lowest AICc (Supplementary Table 3 and Table 1 in Ref. [1] for LRT).

FEL (fixed-effects likelihood), REL (random-effects likelihood), and SLAC (single-likelihood ancestor counting) methods were simultaneously applied to. This server is also based on a site-model calculated with the ML approach [11], [12], [13], [14]. dN/dS > 1 is defined as positively selected site here with statistical confidence (p-value < 0.10 in FEL and SLAC; Bayes Factor > 50 in REL) by testing whether dN is significantly different from dS [11]. The Codon positions detected in dataset 1 are presented in Supplementary Tables 4–6. MSA colored with dN/dS value are presented in Fig. 2 for 13 representative species and for all 64 MSA species in Supplementary Table 7. Next, the ancestral sequence reconstruction was conducted in MEGA7 [3]estimating the maximum likelihood with the MSA and CULT domain phylogenetic tree of dataset 1. Fig. 3 represents the ancestral state of codon 366, detected as positively selected site.

### 2.4 Co-evolution analysis of dataset 2

**Fig. 4:**
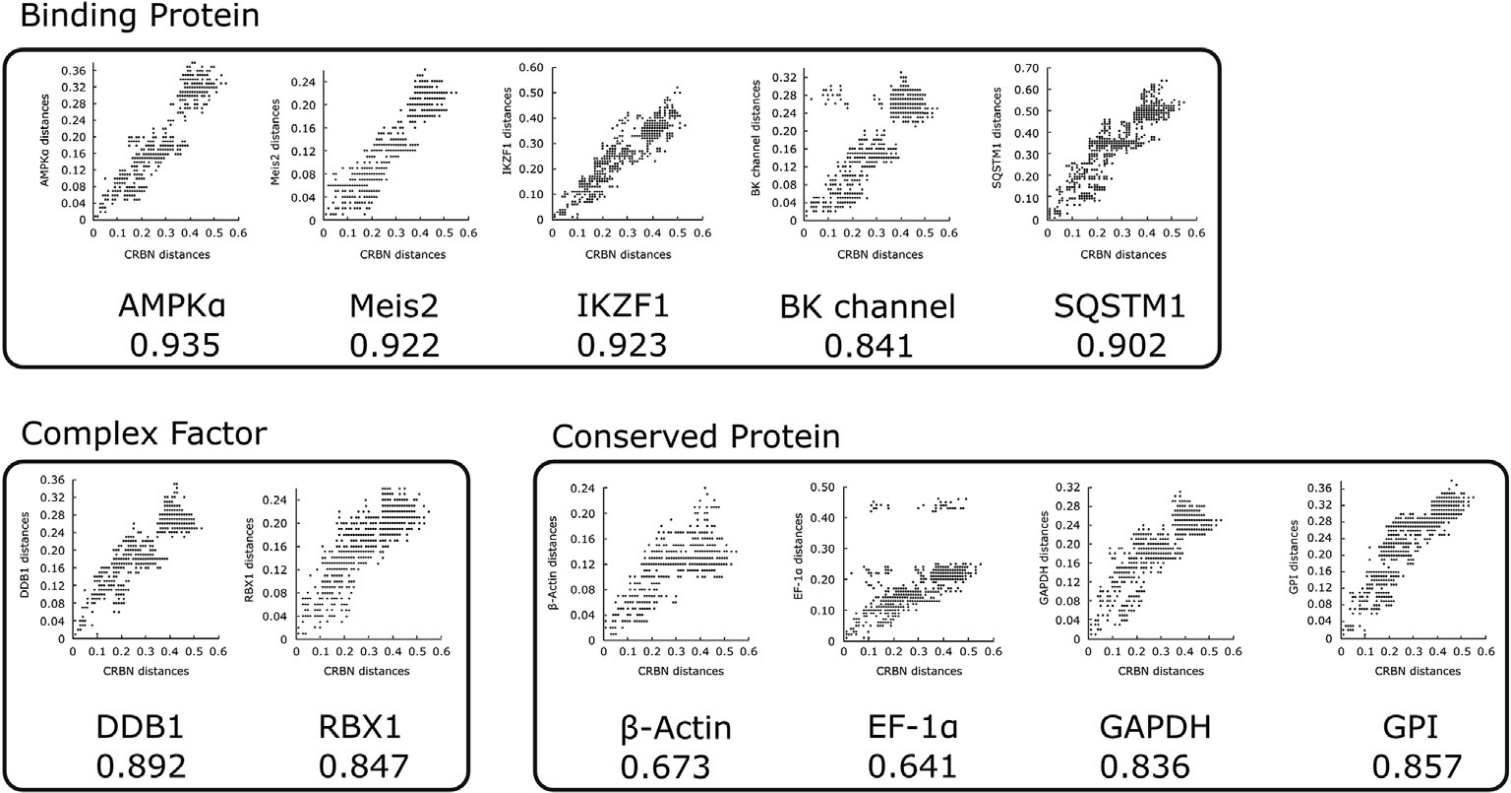
**Correlation coefficient between CRBN and related proteins.** The correlation coefficient was calculated using the MirrorTree Server. The plots show the difference between the corresponding branches of two reconstructed phylogenetic trees.

The protein coding sequences of 11 vertebrate genes were collected from GenBank [2] in May 2018 (Supplementary Table 1). Proteins that are known to be the E3 complex factor or binding partners of CRBN were selected. Here, binding domain of CRBN is not restricted to CULT domain but also Lon domain. Those are DDB1: DNA damage-binding protein1, Rbx1: RING-box protein 1, AMPKα: AMP-activated protein kinase α, IKZF1: IKAROS family zinc finger 1, Meis2: Meis Homeobox 2, SQSTM1: Sequestosome 1, BK channel: Big potassium channel. Four conserved proteins were selected as negative control, GAPDH: Glyceraldehyde-3-phosphate dehydrogenase, GPI: Glucose-6-phosphate isomerase, EF-1α: Elongation factor 1α, and β-Actin. CULT domain, Lon domain, and full length CRBN were separately prepared for comparison between the domains. Partial sequences were cut from the dataset. The sequences were aligned with ClustalW implemented in MEGA7 [3]. Default parameters were used for ClustalW. Redundant sequences were removed manually after multiple sequence alignment, which consisted of a total number of 47-55 sequences for further analysis (Supplementary Table 1). The composition of the sequence species are briefly described in supplementary table 2.A phylogenetic tree was reconstructed with the neighbor-joining method using the maximum composite likelihood model with 500 bootstrap replicates. The trees were uploaded for a co-evolution analysis to the MirrorTree Server [15]. Briefly, the server generates scatter plots from a pair of corresponding species branch lengths of two phylogenetic trees. Then, correlation coefficients, which represent the similarity of evolutionary pressure from two phylogenetic trees, were derived from the plots. For test of significant difference between Lon and CULT domain, p-value was calculated after z-transformation of correlation coefficient. Fig. 4 shows 11 scatter plots derived from CRBN and its related proteins with the respective correlation coefficients. Within the 11 proteins, CRBN-related proteins (E3 complex factors and binding partners) tends to have higher correlation coefficient compared to conserved proteins with statistically significant value for AMPKα (GPI used in statistical comparison) [1]. Furthermore, domain-specific co-evolution analysis is shown in Table 1, exhibiting larger Lon domain's correlation coefficient compared to that of CULT domain for CRBN-related proteins, while no inter-domain difference was observed for conserved proteins.
